# Metabolic changes induced by DNA damage in Ramos cells: exploring the role of mTORC1 complex

**DOI:** 10.1002/2211-5463.13436

**Published:** 2022-05-20

**Authors:** Marcos Castro‐Guarda, Yennyfer Arancibia, Carina Chipón, Christofer Matamala, Paola Oyarzo, Gabriela Vargas, Alejandro Reyes, Mónica Salas, Francisco J. Morera, Angara Zambrano

**Affiliations:** ^1^ 28040 Facultad de Ciencias Instituto de Bioquímica y Microbiología Universidad Austral de Chile Valdivia Chile; ^2^ 28040 Universidad Austral de Chile Coyhaique Chile; ^3^ 28040 Facultad de Ciencias Veterinarias Instituto de Farmacología y Morfofisiología Universidad Austral de Chile Valdivia Chile; ^4^ 28040 Center for Interdisciplinary Studies on the Nervous System (CISNe) Universidad Austral de Chile Valdivia Chile

**Keywords:** double‐strand breaks, etoposide, metabolism, mTOR, rapamycin

## Abstract

DNA damage induces the activation of many different signals associated with repair or cell death, but it is also connected with physiological events, such as adult neurogenesis and B‐cell differentiation. DNA damage induces different signaling pathways, some of them linked to important metabolic changes. The mTORC1 pathway has a central role in the regulation of growth processes and cell division in response to environmental changes and also controls protein synthesis, lipid biogenesis, nucleotide synthesis, and expression of glycolytic genes. Here, we report that double‐strand breaks induced with etoposide affect the expression of genes encoding different enzymes associated with specific metabolic pathways in Ramos cells. We also analyzed the role of mTOR signaling, demonstrating that double‐strand breaks induce downregulation of mTOR signaling. Specific inhibition of mTORC1 using rapamycin also induced changes in the expression of metabolic genes. Finally, we demonstrated that DNA damage and rapamycin can regulate glucose uptake. In summary, our findings show that etoposide and rapamycin affect the expression of metabolic genes as well as apoptotic and proliferation markers in Ramos cells, increasing our understanding of cancer metabolism.

## Graphical abstract

Double‐strand breaks, induced by etoposide, affect the expression of genes encoding different metabolic enzymes. These stimuli also induce downregulation of mTOR signaling, and the specific inhibition of mTORC1 induced changes in the expression of specific metabolic genes. Additionally, etoposide and rapamycin affect the expression of apoptotic and proliferation markers. Our work increases our understanding of cancer metabolism.

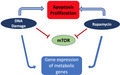

**Abbreviations**
4EBP1: eukaryotic translation initiation factor 4E‐binding protein 1
*ACSL3*
: acyl‐CoA synthetase for long‐chain fatty acids of family 3
ATM: ATM serine/threonine kinase
*BCL2*
: *B‐cell lymphoma‐2*
DDR: DNA damage response
DSB: double‐strand breaks
*FAS*
: Fas *cell surface death receptor*
*G6PD*
: glucose 6‐phosphate dehydrogenase
*GLUT1*
: glucose transporter 1
*GLUT3*
: glucose transporter 3
*HIF1*
: hypoxia 1 inducible factor
*HK2*
: hexokinase 2
*KI67*
: *Antigen* KI‐67
*LDH*
: lactate dehydrogenase
*MTAF*
: mitochondrial transcription factor A
mTOR: mechanistic target of rapamycin
*MVK*
: mevalonate kinase
p70S6K: ribosomal protein S6 kinase beta‐1
PARP: poly (ADP‐ribose) polymerase
*PCNA*
: proliferating cell nuclear antigen
*PDH*
: pyruvate dehydrogenase
*PDK1*
: pyruvate dehydrogenase kinase 1
*PFKP*
: phosphofructokinase
*PGD*
: phosphogluconate dehydrogenase
PPP: Pentose phosphate pathway
*RPE*
: ribulose phosphate 3‐epimerase
*RPIA*
: ribose 5‐phosphate isomerase A
*SC5D*
: sterol‐C5‐desaturase
*SCD1*
: stearoyl‐CoA desaturase

Double‐strand breaks (DSB) are considered among the most cytotoxic damage on the DNA, inducing an essential set of molecular mechanisms to prevent and repair these breaks. The DSB induce the DNA damage response (DDR), which promotes cell cycle arrest, DNA repair mechanisms, and even induces apoptosis and cellular senescence [1]. Interestingly, this DNA repair machinery can be induced in different cells in an intentional and calculated way to generate genetic modification with diverse functionalities, such as neural development, and adult neurogenesis. Specifically, in B cells, the DDR induces B‐cell differentiation [2, 3], where the transcriptional repressor BCL6 regulates this process avoiding cellular transformation, but any genetic or epigenetic alteration may induce cancer, such as B‐cell lymphomas.

DNA damage is associated with metabolic changes, increasing the nucleotide synthesis and anabolic glucose metabolism, and reducing glutamine anaplerosis [4, 5]. However, there is little information about the signaling pathway induced by DSBs and the metabolic changes triggered by these breaks in B cells.

One of the most critical metabolic regulators in many cells is the mechanistic target of rapamycin (mTOR), which is a serine/threonine kinase activated by growth factors, nutrients, and amino acids, which controls fundamental cellular processes such as cell growth, protein synthesis, aging, autophagy, survival, and metabolism [6, 7]. mTOR forms two distinct multi‐protein complexes; mTOR complex 1 (mTORC1) and mTOR complex 2 (mTORC2), containing mTOR, Raptor, mLST8 (GβL), PRAS40, and Deptor; and mTOR, Rictor, Sin1, mLST8, Deptor, and PROTOR, respectively [8, 9]. The activation of mTORC1 induces the phosphorylation of two principal translational regulators, S6K and 4EBP1, to increase ribosome biogenesis and protein synthesis. The activation of mTORC2 regulates the actin cytoskeleton, proliferation, and survival. mTORC1 is sensitive to acute rapamycin‐induced inhibition, while mTORC2 is insensitive to this treatment, but several evidences suggest that a prolonged rapamycin treatment could inhibit mTORC2 in several cell lines [10, 11].

mTORC1 signaling has been associated with nucleotide synthesis, specifically promoting *de novo* pyrimidine and purine synthesis through several mechanisms [12, 13], suggesting that this pathway is also crucial in the regulation of another significant metabolic pathway, the pentose phosphate pathway (PPP). mTORC1 also appears to play a crucial role in lipid synthesis, inducing the activation of lipogenic gene expression and increasing lipid synthesis [14, 15]. Besides, the mTORC1 pathway induces glycolysis and glutaminolysis processes to support anabolic functions, inducing the expression of glucose transporters and many other enzymes associated with these pathways as well [16].

In the immune system, mTORC1 has been connected to T‐ and B‐cell differentiation, migration, tolerance, and antibody production [17, 18, 19, 20]. In B cells, the activation of mTORC1 results in an increase in glucose uptake and ribosomal biogenesis [21]. Indeed, this pathway is activated in positively selected B cells, inducing an increase in anabolic pathways; in the same way, decreases in mTORC1 activity are related to cessation of proliferation [22]. Even when, in B cell, mTORC1 signaling is critical for establishing an effective Ab production, there is no evidence whether DNA damage associated with these events is related to mTORC1 signaling.

The principal aim of this work was to evaluate the metabolic changes induces by etoposide, which induces DSB in Ramos cells. We determined whether DSB can regulate mTORC1 signaling. Also, we analyzed the effect of the direct inhibition of the mTORC1 pathway on metabolism pathways using rapamycin. Interestingly, the effect of DSB and rapamycin on metabolic gene expression was similar in several genes, suggesting that mTORC1 inhibition could be involved in regulating these genes when DSBs occur. Additionally, we analyzed the effect of DSB and the inhibition of mTORC1 on survival processes. DBS and rapamycin present significant differences in the modulation the expression of several genes associated with cell apoptosis and cell proliferation, suggesting that DBS requires different signaling pathways to promote these events. These results provided important information about DSB molecular mechanism in association with mTOR pathway.

## Materials and methods

### Cell culture

Ramos cells are a B lymphocyte cell line from Burkitt’s Lymphoma (ATCC CRL‐1596™). This cell line was grown in RPMI‐1640 (from Corning) supplemented with 10% fetal bovine serum (from Biowest, FL, USA), 100 U·mL^−1^ penicillin, 100 mg·mL^−1^ streptomycin, and 2 mm l‐glutamine (from Hyclone, Logan, UT, USA), 1 mm sodium pyruvate, and essential amino acids (from Corning, Glendale, AZ, USA), at 37 °C in humidified 5% CO_2_ atmosphere.

### Western blot analysis

Cells were cultured and treated with 20 µm etoposide (from Sigma, Darmstadt, Germany) or 50 nm rapamycin (Calbiochem, San Diego, CA, USA) for different periods. Then, cells were lysed in lysis buffer (50 mm Tris–HCl pH 7.4; 150 mm NaCl; 1 mm EDTA; 1% Triton X‐100, 1 mm Sodium orthovanadate, 100 mg·mL^−1^ PMSF, 2 mg·mL^−1^ aprotinin, 2 mm leupeptin, and 1 mg·mL^−1^ pepstatin), and protein concentration was determined using the Bradford assay. Protein extracts were resolved by SDS/PAGE, 100 or 60 μg per lane, on a 6% or 12% polyacrylamide gels and transferred into Nitrocellulose Membrane, 0.45 µm (Bio‐Rad Laboratories, CA, USA). Next, the membrane was blocked with 5% bovine albumin serum or 5% skimmed milk, and then membranes were incubated with 1 : 5000 dilutions of primary antibodies against p‐4EBP1, p‐P70S6K, p‐mTOR, or total proteins to evaluate the mTOR pathway (Cell Signaling Technology, Inc., Danvers, MA, USA; cat No.2855; cat No.9234; cat No.5536, respectively). Cleaved caspase 3 and cleaved PARP (Cell Signaling Technology, Inc.; cat No.9661, cat No. 5625, respectively) to determine apoptosis marker. Antibodies against p‐ATM and total ATM to evaluate DNA damage (Cell Signaling Technology, Inc.; cat No.4526 and cat No.2873, respectively). Tubulin (Calbiochem, Darmstadt, Germany, cat No. CP06) was used as loading control.

### Quantitative real‐time reverse transcriptase‐polymerase chain reaction (RT‐qPCR)

Total RNA was isolated from cells using Trizol reagent (Life Technologies, Waltham, MA, USA) following the manufacturer’s instructions. Total RNA was subjected to RT‐PCR. 1–5 µg of total RNA was used to synthesize first‐strand cDNAs with the iScript kit (Bio‐Rad, CA, USA). Quantitative RT‐PCR (RT‐qPCR) analysis was performed as described previously [23]. Expression was normalized to 36B4 mRNA expression as a housekeeping gene. Oligonucleotide primers for real‐time RT‐qPCR are described in Table A1 in the Appendix section [24].

### Glucose transport assay

Transport assay was performed using a glucose analog, 2‐deoxy‐d‐Glucose (2‐DOG), to measure glucose uptake in treated Ramos cells. The assay was carried out in a final volume of 200 μL, where 30 μL corresponded to a suspension of cells previously treated for different periods of time with etoposide or rapamycin, or control cells using only DMSO, the vehicle. The cell suspension corresponds to approximately 10 million of cells in PBS at pH 7.4, supplemented with 1 mm CaCl_2_ and 0.5 mm MgCl_2_. The remainder (170 μL) corresponds to a radioactive mixture containing 0.5 μCi of 2‐DOG‐[3H], 0.25 mm 2‐DOG at cold and 1× PBS pH 7.4 supplemented 1 mm CaCl_2_ and 0.5 mm MgCl_2_ at 4 °C. To perform the test, 30 μL of cells with 170 L of radioactive mixture was mixed, and after 30 s the reaction was stopped by adding 1 mL of stop solution (PBS 1× pH 7.4 supplemented with 1 mm CaCl_2_ and 0.5 mm MgCl_2_ at 4 °C). For the basal uptake controls at time 0, the stop solution was added to the cells before the radioactive mixture. Cells were collected by centrifugation at maximum speed for 10 s, the supernatant was removed, and the cell pellet was washed with 1 mL of the stop solution, then the cells were centrifuged again under the same conditions, and the supernatant was removed. The resulting pellets were suspended in 200 μL of cell lysis buffer (50 mm Tris–HCl pH 7.4; 150 mm NaCl; 1 mm EDTA; 1% Triton X‐100) and left overnight. The next day the lysate was transferred to a vial, and 2 mL of scintillation fluid was added to the tube. Finally, the radioactivity was measured using a PerkinElmer Tri‐Carb 2910 TR counter (OH, USA). To determine whether the glucose uptake by Ramos cells was dependent on the GLUTs transporters, we analyze the effect of cytochalasin B. Briefly, cytochalasin B was added to the radioactive mixture at a final concentration of 1 μm.

### Cell viability assay using propidium iodide

Cells were cultured in a 96‐well plate in 100 μL of RPMI‐1640 without phenol red (from Corning), supplemented with 10% fetal bovine serum (from Biowest), 100 U·mL^−1^ penicillin, 100 mg·mL^−1^ streptomycin and 2 mm l‐glutamine, (from Hyclone), 1 mm Sodium Pyruvate, and essential amino acids (from Corning), at 37 °C in humidified 5% CO_2_ atmosphere. After 24 h, the cells were treated with different concentrations of etoposide or rapamycin. *N*,*N*‐dimethylformamide was used as a positive death control. Once the treatment times were over, it was added 100 µL of HBSS modified‐Ca^+2^ (40 µm KH_2_PO_4_; 30 µm NaH_2_PO_4_xH_2_O; 13.6 µm NaCl; 600 µm d‐glucose; 500 µm KCl; 900 µm CaCl_2_) with propidium iodide (from Thermo Fisher Scientific, Eugene, OR, USA) to a final concentration of 5 µm per well. On the contrary, in control death wells, 2 µL from a 500 µm propidium iodide stock was added. The plates were incubated at 37 °C for 5 min, and the plate was measured on a fluorescent plate reader using an excitation wavelength of 530 nm and an emission wavelength of 620 nm.

### Statistical analysis

Data are presented as mean ± SD in the case of qPCR, western blot, and viability plots, and as mean ± SE and in the case of radioactive transport plots of the values from the number of experiments performed in triplicate. Data were analyzed for statistically significant differences using the Mann–Whitney test (Student’s *t*‐test) or ANOVA (Tukey’s test) as indicated in the corresponding figures. The data were graphed and analyzed using the graphpad prism 6 program (San Diego, CA, USA).

## Results

### DNA damage modulates the expression of metabolic genes in Ramos cells

**Fig. 1:**
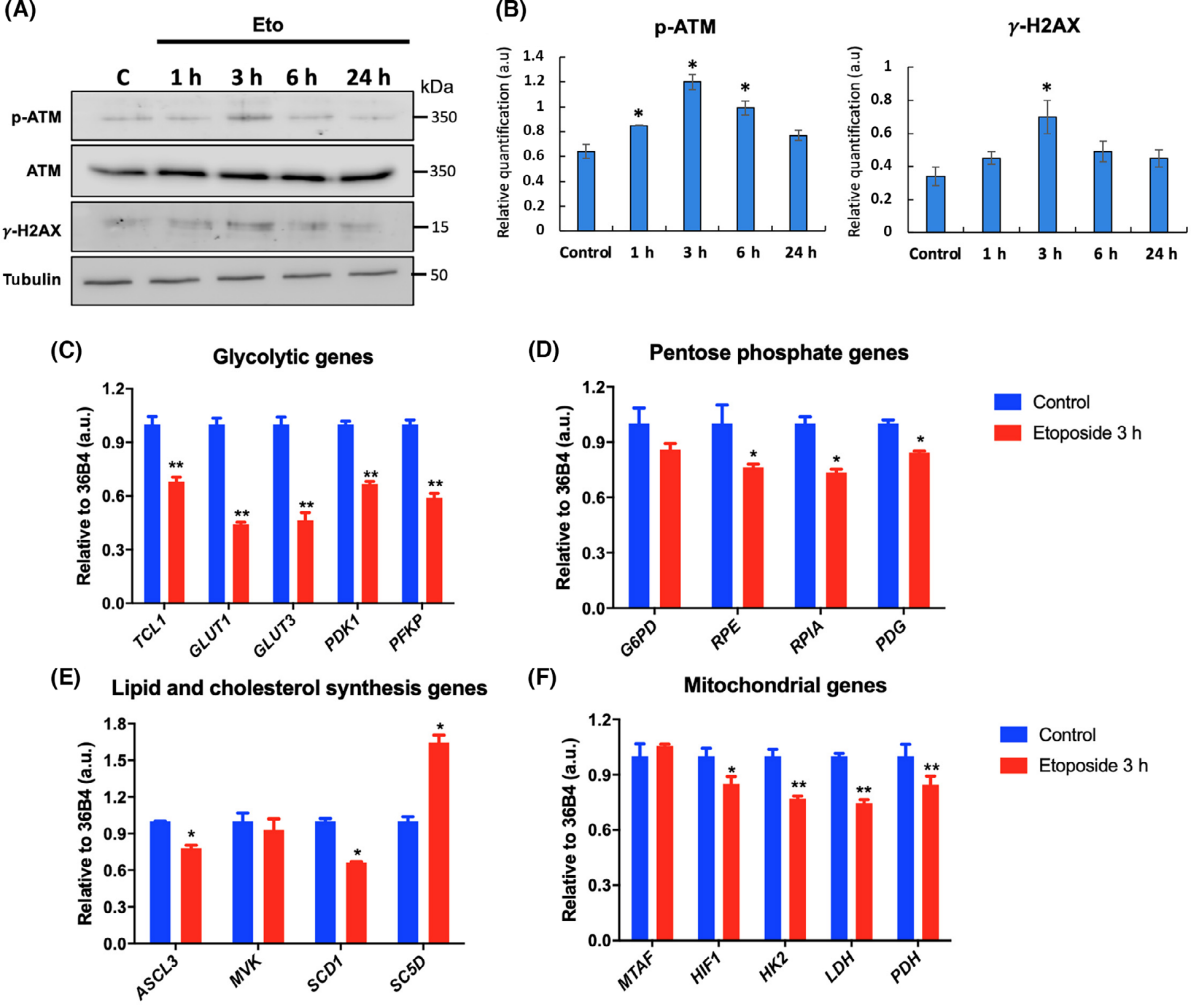
Etoposide modulates the expression of metabolic genes. (A) Western blotting of total Ramos cell proteins treated with 20 µm etoposide for 1, 3, 6, and 24 h, using anti‐p‐ATM, ATM, γ‐H2AX, and tubulin antibodies to evaluate DNA damage. (B) Densitometry quantitation analysis of western blot assay, the result was expressed as the ratio of p‐ATM levels with respect to total ATM, or γ‐H2AX levels to tubulin. Data are presented as mean ± SE. Then, the Ramos cell line was treated with 20 µm etoposide for 3 h. To analyze its effect on metabolic gene expression. (C) Effect on glycolytic gene expression. (D) Pentose phosphate pathways genes. (E) Lipid and cholesterol synthesis genes. (F) Mitochondrial genes. 36B4 was used as a housekeeping gene, and DMSO was used as vehicle. Error bars indicate SD, *P*‐values determined by Mann–Whitney test (nonparametric *t*‐test). **P < 0.001; *P < 0.01. The results are representative of three independent experiments.

To evaluate the effect of DSB on gene transcripts level of the different metabolic pathways, we used etoposide and RT‐qPCR assays to analyze the expression of different genes that actively participate in glucose metabolism, pentose phosphate pathway, lipid and cholesterol synthesis, and some mitochondrial genes [24]. First, to test DNA damage induced by etoposide, we treated Ramos cells with this compound during different times, and we analyzed the phosphorylation of an important DNA damage sensor such as ATM and the increase in γH2AX. In Fig. 1A,B, we can see that 20 μm of etoposide can induce a clear increase in ATM phosphorylation levels at 3 h, suggesting that this drug can induce DNA damage in this model; also there is a significative increase in the levels of γH2AX at the same time point. These markers decrease after 6 h treatment. Then, the Ramos cells were treated with etoposide for 3 h; etoposide induces significant changes in the expression of several genes associated with proliferation at this time [2]. In respect to glucose metabolism genes (Fig. 1C), we evaluated the expression of glucose transporter 1 (*GLUT1*), glucose transporter 3 (*GLUT3*), pyruvate dehydrogenase kinase 1 (*PDK1*), and phosphofructokinase (*PFKP*). A significant decrease in the transcript levels was seen after inducing DNA damage in all these genes, highlighting that the most affected genes were *GLUT1* and *GLUT3*, which decreased at least 50%. As a control, we analyzed the expression of *TCL1* gene because it has been previously demonstrated that etoposide decreases the expression of this gene at 20 µm for 3 h of treatment in Ramos cells.

A similar effect was observed in the expression of genes associated with pentose phosphate pathways (Fig. 1D), where the expression of glucose 6‐phosphate dehydrogenase (*G6PD*), ribulose phosphate 3‐epimerase (*RPE*), ribose 5‐phosphate isomerase A (*RPIA*), and phosphogluconate dehydrogenase (*PGD*) genes were evaluated. In this case, the treatment with etoposide induced a significant decrease in at least 20% in the transcript levels of the *RPE*, *RPIA*, and *PGD* genes, while the expression of the *G6PD* gene had no variation with respect to the control cells.

To analyze the genes related to the synthesis of lipids and cholesterol (Fig. 1E), the expression of acyl‐CoA synthetase for long‐chain fatty acids of family 3 (*ACSL3*), mevalonate kinase (*MVK*), stearoyl‐CoA desaturase (*SCD1*), and sterol‐C5‐desaturase (*SC5D*) genes were evaluated. In this case, the treatment with etoposide was differentially modulating the expression of these genes. To *ACSL3* and *SCD1* gene expression, we saw a significant decrease in at least 20% in their transcript levels, while the *MVK* gene expression did not show variations with respect to the control. Interestingly, the expression of the *SC5D* gene increased significantly by approximately 70% with the treatment.

Regarding the mitochondrial genes (Fig. 1F), mitochondrial transcription factor A (*MTAF*), hypoxia 1 inducible factor (*HIF1*), hexokinase 2 (*HK2*), lactate dehydrogenase (*LDH*), and pyruvate dehydrogenase (*PDH*) were evaluated. There was a significant decrease of at least 10% in the transcript levels of the *HIF1*, *HK2*, *LDH*, and *PDH* genes, while there were no significant variations in *MTAF* gene expression.

Overall, our results demonstrate that etoposide can modulate the expression of various genes associated with different metabolic pathways in Ramos cells.

### Etoposide inhibits mTOR signaling pathway

**Fig. 2:**
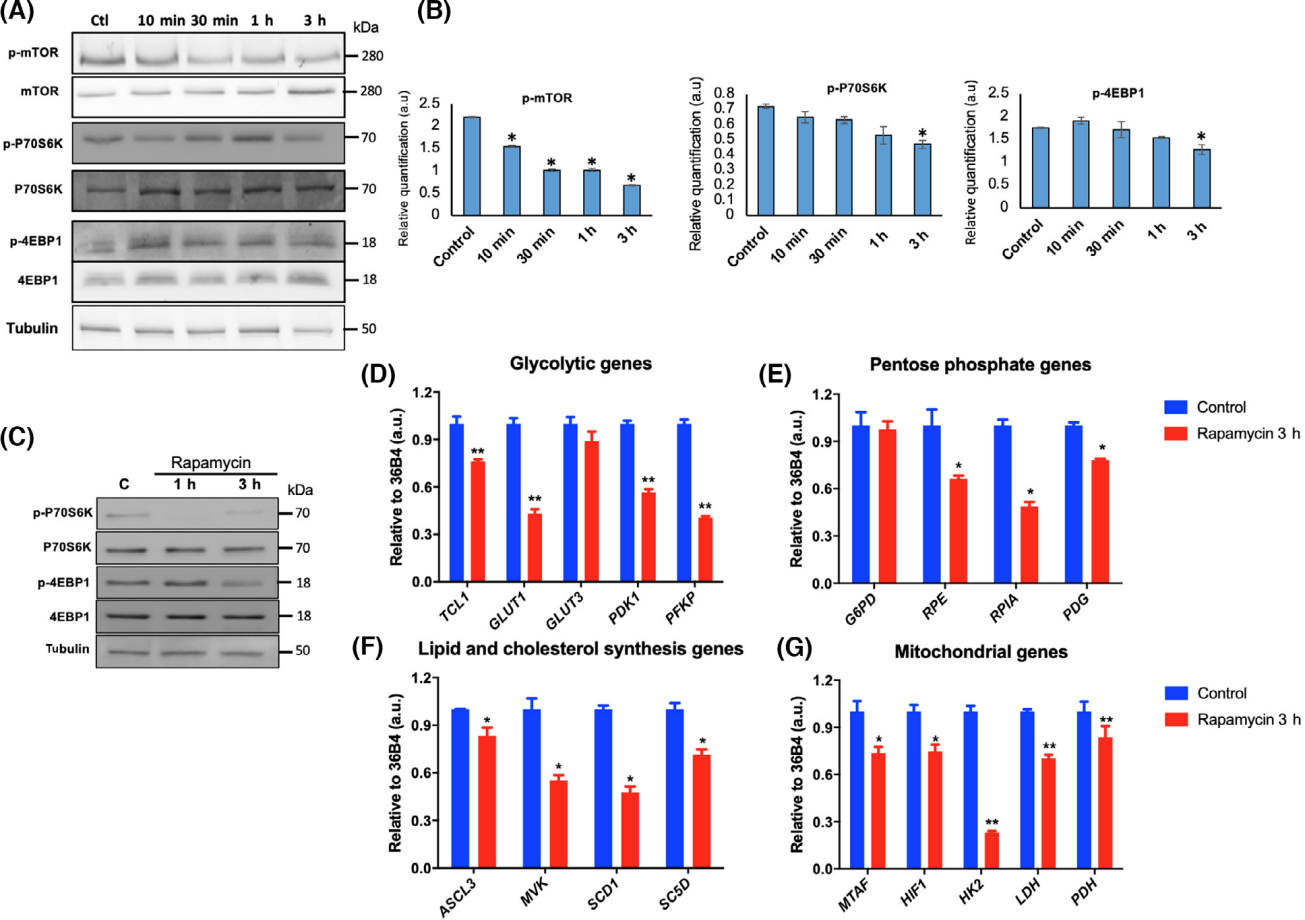
Etoposide modulates phosphorylation levels of the mTOR pathway, and rapamycin modulates the expression of metabolic genes. (A) Western blot analysis of total Ramos cell proteins treated with 20 µm etoposide for periods of 10 min to 3 h, using specific antibodies to evaluate mTOR pathway. (B) Densitometry quantitation analysis of western blot assay, for each case the result was expressed as the ratio of phosphor‐protein levels with respect to total protein. Data are presented as mean ± SE (C) Ramos cell line was treated with 50 nm rapamycin for 1 and 3 h. Immunoblot analysis was performed using specific antibodies to evaluate mTOR pathway. For qPCR assay, the cells were treated with 50 nm rapamycin for 3 h. (D) Effect on glycolytic gene expression. (E) Pentose phosphate pathways genes. (F) Lipid and cholesterol synthesis genes. (G) Mitochondrial genes. 36B4 was used as a housekeeping gene, and DMSO was used as vehicle. Error bars indicate SD, *P*‐values determined by Mann–Whitney test (nonparametric *t*‐test). **P < 0.001; *P < 0.01. The results are representative of three independent experiments.

Since mTOR signaling has a vital role in metabolic changes, we evaluate the effect of DSB, induced by etoposide, on the phosphorylation state of mTOR and some classical targets of the mTOR signaling pathway in Ramos cells. For this event, we measured the level of mTOR phosphorylated on Ser 2448, a key site for kinase activity [25], and the phosphorylation levels of two principal mTORC1 targets: (a) P70S6K in its residue Thr389, which is the key of its kinase activity [26] and (b) 4EBP1 in its Thr37/46 residues, which should be noted, do not prevent the binding of 4EBP1 to eiF4E, but allow subsequent phosphorylation of other sites which are associated with the initiation of translation process [27]. As shown in Fig. 2A,B, our results demonstrated that the treatment with etoposide induces a decrease in mTOR phosphorylation levels in a time‐dependent way. Analyzing the specific target of mTOR; P70S6K, the treatment with etoposide also induces a decrease in P70S6K phosphorylation levels. Concerning 4EBP1, in this case, the etoposide has a low effect on 4EBP1 phosphorylation levels, inducing a significant decrease only at 3 h post‐treatment. These results suggest that etoposide induces a decrease in phosphorylation degree on mTORC1 targets, suggesting that this drug can decrease the activity of this pathway in Ramos Cells.

### Rapamycin modulates the expression of metabolic genes in Ramos cells

Since that etoposide induced a clear decrease in mTORC1 signaling, we proceeded to evaluate the expression of the same pool of metabolic genes by RT‐qPCR, but using cells treated with 50 nm of rapamycin, an inhibitor of mTOR. First, to analyze the effect of rapamycin in these cells, we study mTOR signaling pathway under rapamycin treatment. As shown in Fig. 2C, our results demonstrated that the treatment with rapamycin induces a decrease in phosphorylation levels of two classical targets of mTORC1. The treatment with rapamycin induces a clear decrease in P70S6K phosphorylation levels. Concerning 4EBP1, in this case, the rapamycin induced a decrease in 4EBP1 phosphorylation after 3‐h treatment. These results demonstrate that rapamycin induces a clear inhibition of mTORC1 signaling in Ramos Cells.

With respect to the genes associated with glucose metabolism (Fig. 2D), the behavior is similar to that observed with etoposide (Fig. 1C), with a significant decrease of at least 20% in transcript levels of *TCL1* and a decrease at least 40% of all glycolytic genes evaluated, except for the *GLUT3* gene, that in this case does not show variations in its expression with respect to the control.

Regarding the genes of the pentose phosphate pathway (Fig. 2E), there was no variation in the expression of the *G6PD* gene with respect to the control. A significant decrease in the *RPE*, *RPIA*, and *PDG* genes was seen; however, the decrease in these genes´ transcript levels was more significant with rapamycin than etoposide.

In the case of genes of lipid and cholesterol synthesis (Fig. 2F), the behavior was different from that observed with etoposide (Fig. 1E) in most of the genes analyzed. Although the transcript levels of the *ACSL3* and *SCD1* genes showed the same tendency as in the previous case, the decrease in the *SCD1* gene expression was more significant with rapamycin by approximately 50% than with etoposide. On the contrary, a significant decrease of approximately 40% in the *MVK* gene expression was observed with rapamycin, but not with etoposide. The most surprising change was in the transcript levels of the *SC5D* gene, which increase its expression with etoposide, but showed a clear decrease of approximately 30% with rapamycin.

In the case of genes associated with mitochondrial function (Fig. 2G), the treatment with rapamycin also induced similar results than etoposide, with some exceptions (Fig. 1F). In this case, the treatment with rapamycin induces a significant decrease of approximately 20% in *MTAF* gene expression. Concerning *HIF1* and *HK2* genes, rapamycin treatment induces a remarkable decrease in gene expression, especially for the *HK2* gene, where the expression decreased approximately 70%. Regarding the *LDH* and *PGD* genes, the expression of both genes decreased in the presence of rapamycin.

Overall, our results suggest that rapamycin treatment showed similar behavior to etoposide treatment on the gene expression of several genes analyzed.

### Etoposide and rapamycin affect glucose uptake, mediated by GLUTs

Although a similar effect has been observed in the treatments of etoposide and rapamycin on the expression of different genes of the metabolic pathways, it is essential to know whether these drugs have a similar effect at the functional level, specifically glucose transport, which is necessary for the proliferation and differentiation processes, principally due to the decrease in *GLUT1* transcript levels observed previously (Figs 1 and 2).

**Fig. 3:**
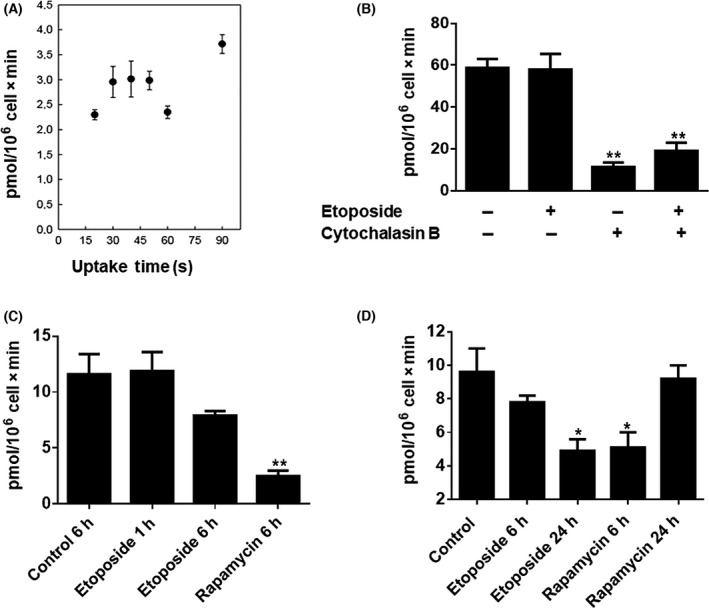
Effect of etoposide and rapamycin on DOG uptake. Ramos cell line was incubated with 20 µm etoposide or 50 nm rapamycin for different hours, and 0.5 µCi of 2‐DOG‐[3H] was added for 15 s in Panel B and 30 s in Panel C and D. (A) The DOG uptake was measured in Ramos cell at different times. (B) Effect of cytochalasin B in DOG uptake in cells incubated with or without etoposide; in this case, the cells were treated using 20 μm etoposide for 3 h. (C) Effect on the DOG uptake in cells treated with etoposide or rapamycin in early times. (D) Effect on the uptake in cells treated with etoposide or rapamycin in early and late times. The results are representative of three independent experiments. Error bars indicate SD, *P*‐values determined by ANOVA. **P < 0.001; *P < 0.01.

The glucose uptake was measured in Ramos cells using a non‐metabolizable glucose analog, 2‐DOG, radioactively labeled. The uptake was measured at different periods. These kinetic analyses show that the glucose incorporation rate was linear until 30 s (Fig. 3A). The next step was to assess whether the transport of glucose from Ramos cells was mediated primarily by facilitative glucose transporters, GLUTs (Fig. 3B). For this aim, we treated Ramos cells only with the vehicle, DMSO, or with 20 µm etoposide for 3 h, and we used cytochalasin B (CCB), a well‐known inhibitor of GLUTs transporters. As we can see, the treatment with etoposide does not change the glucose analog transport at 3 h with respect to the control. However, in both cases, control cells or treated with etoposide, the incubation with CCB induce a significant decrease, in approximately 80% and 70%, respectively, in the uptake of 2‐DOG. These results indicate the high dependence of this process on the GLUTs transporters in Ramos cells.

Subsequently, to evaluate the effect of etoposide or rapamycin on glucose uptake in Ramos cells, we treated the cells with 20 µm etoposide or 50 nm rapamycin for different periods. The effect was evaluated both early (Fig. 3C) and late (Fig. 3D) times of treatment. Our results show a clear difference in the effect of both drugs. Regarding the early times, as can be seen in treatments of 1 and 6 h (Fig. 3C) and 3 h (Fig. 3B) of etoposide does not affect glucose analog uptake. In contrast, 6 h of treatment with rapamycin induced a significant decrease in glucose transport of these cells of approximately 80%. However, the treatment at late times (24 h) with this drug; the uptake is restored (Fig. 3D). On the contrary, in late treatment times, etoposide showed a significant decrease in glucose transport, approximately 50%. Our results showed that glucose transporters GLUTs mainly mediate the glucose uptake in Ramos cells, and etoposide and rapamycin affect this metabolite's uptake in Ramos cells differently; rapamycin inhibits the uptake at early times of treatment, and etoposide inhibits the glucose uptake at late times.

### The effect of etoposide and rapamycin on the regulation of apoptosis and proliferation genes

**Fig. 4:**
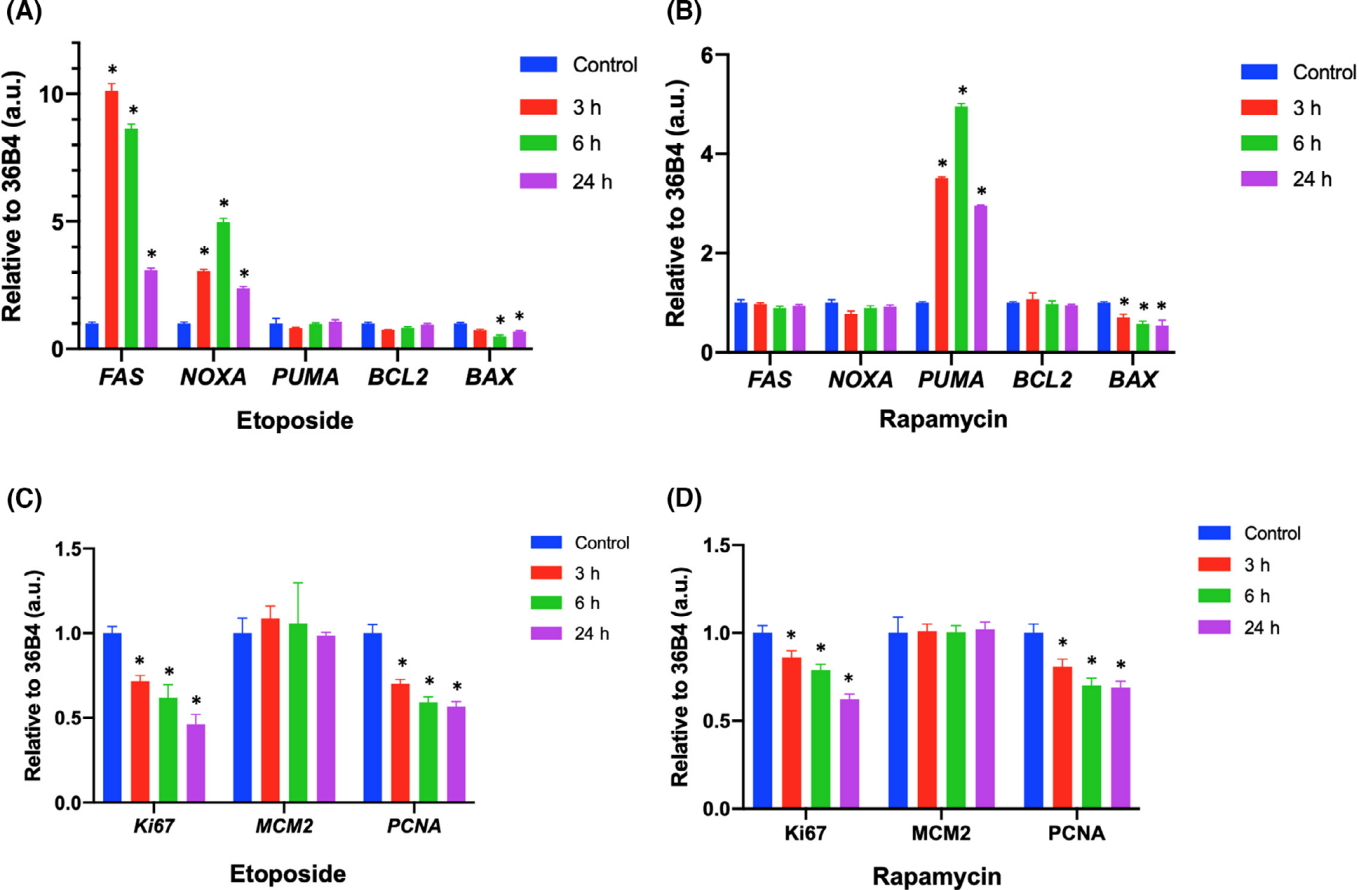
DNA damage and mTORC1 inhibition affect the expression of genes associated with apoptosis and cell proliferation. Ramos cell line was treated with 50 nm rapamycin or 20 µm etoposide for different periods of times. (A) Effect of etoposide on apoptotic gene expression. (B) Effect of rapamycin on apoptotic gene expression. (C) Effect of etoposide on proliferation gene expression. (D) Effect of rapamycin on proliferation gene expression. 36B4 was used as a housekeeping gene and DMSO was used as vehicle. The results are representative of three independent experiments. Error bars indicate SD, *P*‐values determined by Mann–Whitney test (nonparametric *t*‐test). *P < 0.05.

Since the decrease in metabolic rate, especially glucose uptake, is associated with cell death activation, we evaluated the effect of etoposide and rapamycin on the gene expression of several genes associated with apoptosis (Fig. 4A,B) and proliferation processes (Fig. 4C,D). To study the apoptosis cell death, we evaluated the expression of *FAS*, *NOXA*, *PUMA*, *BCL2*, and *BAX* genes. We observed that both etoposide and rapamycin affect the expression of these genes but in a different way. Etoposide triggers a significant increase in the expression of *FAS* and *NOXA* genes (Fig. 4A). In the expression of *FAS*, there was an increase in transcription levels of approximately eleven times greater than the control at 3 h. For *NOXA*, there was an increase of five times at 6 h. On the contrary, rapamycin treatment showed a clear increase of approximately five times in the *PUMA* gene expression at 6 h (Fig. 4B). Regarding the *BCL2* gene, there are no differences with respect to the control with any treatments, and the results related to the *BAX* gene show a significant decrease in both treatments at all the times analyzed (Fig. 4A,B).

Then, *KI67*, *MCM2*, and *PCNA* proliferation genes were analyzed; in this case, it was detected that both treatments affect the expression of these genes in a very similar manner (Fig. 4C,D), showing that there was no variation in the levels of transcription for the *MCM2* gene with respect to the control, but we observed a significant decrease in the transcripts of *KI67* and *PCNA* with both drugs.

Thus, etoposide and rapamycin modulate apoptosis genes differently, each having specific targets but regulating proliferation genes in a similar inhibitory manner.

### The effect of etoposide and rapamycin on cell viability

**Fig. 5:**
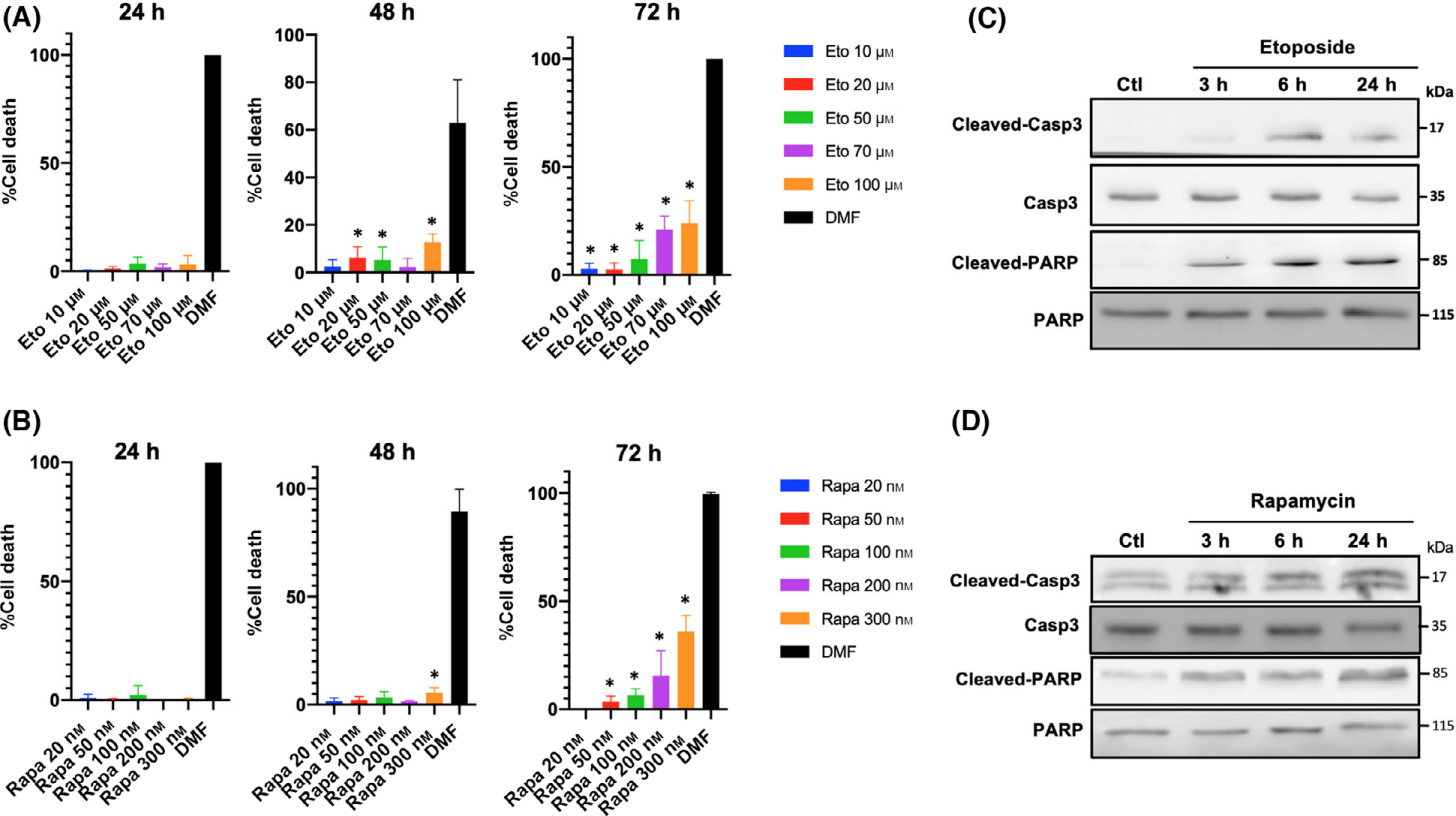
Effect of etoposide and rapamycin on cell viability. Ramos cell line was treated with different concentrations of etoposide or rapamycin at different time‐periods. (A) Effect of etoposide on the cell viability after 24, 48, and 72 h of treatment. DMSO was used as negative control and DMF (25% Dimethylformamide and 25% SDS) as positive death control. (B) Effect of rapamycin on the cell viability after 24, 48, and 72 h of treatment. DMSO was used as negative control and DMF (25% Dimethylformamide and 25% SDS) as positive death control. (C) Effect of etoposide 20 μm on cleaved Caspase 3 and cleaved PARP levels. (D) Effect of rapamycin 50 nm on cleaved Caspase 3 and cleaved PARP levels. These results are representative of three independent experiments. The results are representative of three independent experiments. Data are presented as mean ± SE, *P*‐values determined by ANOVA. *P < 0.05.

Etoposide and rapamycin induce evident variations in the expression of different genes associated with the apoptosis process, suggesting that these stimuluses induced cell death. To test this hypothesis, viability assays were carried out using propidium iodide at 24, 48, and 72 h of treatment. As we can see in Fig. 5A, the treatment with etoposide significantly affects Ramos cells viability, increasing cell death by approximately 10% with concentrations of 20 µm at 48 h of treatment and 30%, with concentrations of 100 µm of this drug at 72 h of treatment. The results show that cell death is time and concentration dependent. Using different concentrations of rapamycin, we could see that this drug also can induce an increase in cell death, which was significant using 300 nm at 48‐h treatment, and 50 nm or more at 72‐h treatment (Fig. 5B).

Finally, we determined the levels of protein markers associated with apoptosis using etoposide or rapamycin at 3, 6, and 24 h post‐treatment; specifically, evaluating the levels of cleaved caspase 3 and cleaved PARP with both treatments. The treatment with etoposide induces a significant increase in the levels of cleaved caspase 3 at 6 h post‐treatment. Similar results were obtained on the levels of cleaved PARP with a clear increase at 3 h post‐treatment (Fig. 5C). The treatment with rapamycin also induces an increase in cleaved caspase3 and fragmented PARP (Fig. 5D). These results demonstrate that the increase in cell death using etoposide and rapamycin is accompanied by an increase in classical apoptotic markers.

## Discussion

DNA damage induces the activation of several signaling pathways, most of them associated with repairing and cell death; however, this event may induce many other cellular responses. In the current study, we evaluate the metabolic changes during DSB in Ramos cells, analyzing the gene expression profile of enzymes associated with different metabolic pathways. Also, in this work, we evaluated the regulation of mTORC1 pathway induced by etoposide and its possible role in the metabolic changes observed.

Cancer cells or high proliferative cell lines take up more substantial amounts of glucose than normal cells and metabolize it by glycolytic pathway at high rates [28]. In this work, we used the Ramos cell line, a B lymphocyte from Germinal Center (GC). GC is a site of high rate of proliferation associated with the increase in glucose uptake and ribosomal biogenesis [21]. It has been demonstrated that DNA damage, induced by DNA rearrangement, promotes B‐cell differentiation into long‐lived plasma cells and memory B cells [2], as we have known the differentiation process involves downregulation of glycolysis pathway and upregulation of oxidative phosphorylation (oxphos) pathway [29].

We found that DSB induced by etoposide in Ramos cells decreased the expression of several metabolic enzymes associated with glucose metabolism, the pentose phosphate pathway, and some genes associated with mitochondrial function and lipid and cholesterol synthesis. Also, in these cells DNA damage induces similar results in other cell models; specifically, DNA damage induces the activation of several DNA damage sensors like ATM that induces the activation of p53 by phosphorylation [30], which has been directly associated with *GLUT1* and *GLUT4* gene repression. Interestingly, we demonstrated that DSB induces a clear decrease in glucose uptake in this cell model, which is related to the decrease in *GLUT1* and *GLUT3* expression induced by etoposide. Moreover, Poly (ADP‐ribose) polymerase 1 (PARP1), which also acts as a DNA damage sensor and is quickly activated in response to DNA damage [31, 32], has been related to affect metabolism; PARP has also been reported to inhibit hexokinase (HK), a critical enzyme in the glycolytic pathway [33, 34]. It has been demonstrated that PARP activation shifts the metabolic reliance to oxphos, which has been suggested that it is critical for damaged cell survival [31]. Also, it is well known that metabolism impacts DDR pathway via the regulation of metabolite pools; both glycolysis and glutaminolysis promote DSB repair [35]. Our results also have shown a decrease in the expression of several genes associated with the pentose phosphate metabolic pathway, which is crucial for nucleotide synthesis and DNA repair. We could see a decrease specifically for *RPE*, *RPIA*, and *PGD*, but not in *G6PD*. Interestingly, it has been demonstrated that the activation of p53 can inhibit the G6pd activity, which is a limiting enzyme of this pathway [36]. Therefore, it will be essential to determine the effect of DNA damage on the G6pd activity in future. Also, our results provide evidence that DNA damage induces a downregulation in the expression of *ACSL3* and *SCD1*, both involved in fatty acid metabolism, which is often dysregulated in highly proliferative and cancer cells [37, 38], unlike what happens with the case of the *MVK* and *SCD5* enzymes that have not been related to cancer prognosis [39].

There is plenty of evidence demonstrating that mTOR signaling can modulate several metabolic pathways, including glycolysis, pentose phosphate, and lipid biosynthesis [15]. The effect of DNA damage on mTOR signaling in other models has been studied with controversial results [40, 41, 42, 43]. It has been demonstrated that mTOR is a signaling pathway involved in DNA repair. Indeed, there is evidence that the inactivation of mTOR induces the autophagy process and promotes the transcriptional expression of damage‐regulated autophagy modulator (DRAM), which is a lysosomal protein that facilitates the autophagy. Depending on the extent of DNA damage, autophagy plays a cytoprotective or cytotoxic role in cancer.

In this work, we demonstrated that damage induced by etoposide triggers a downregulation of mTOR signaling, inducing a decrease in phosphorylation on mTOR and its classical target; P70S6K in Ramos cells. mTOR is frequently deregulated in human cancer, and indeed mTOR inhibitors have been commonly used to treat human malignancies [21]. It is estimated that between 60% and 80% of all cancers have active mTORC1 signaling. The mTOR is a serine‐threonine kinase critically involved in controlling anabolism and metabolic reprogramming in immune cells [44]. Different stimuli, including glucose, growth factors, and other signals, can activate mTOR complex 1 (mTORC1), which induce cell growth [45]. To analyze the effect of mTORC1 signaling, we use rapamycin as an mTOR inhibitor. As we expect, the effect of rapamycin on the metabolic pathways was very similar to that induced by DNA damage, suggesting that downregulation of mTORC1 is involved for this metabolic switch. However, we demonstrate that etoposide affects *GLUT3* gene expression, but rapamycin does not, suggesting that etoposide and rapamycin probably have a different o parallel mechanism to exert their inhibitory effect on glucose uptake in these cells. Previously, other researchers demonstrated that etoposide induces a decrease in glucose uptake by Hodgkin's lymphoma cells possible through downregulating glucose transporters [46]; also, there has been described that genotoxic exposure inhibits the expression of both GLUT1 and GLUT3 genes [47], Similar discovers were described for Rapamycin, specifically rapamycin reduces glucose uptake in human adipocytes [48], and in CTLs cells rapamycin caused an approximate 50% reduction in levels of GLUT1 and GLUT3 [49].

When we assess the effect of etoposide and rapamycin on cell viability, both treatments induced a significant decrease in this parameter. Our results suggest that etoposide induces an increase in the expression of apoptosis‐associated proteins such as *FAS* and *NOXA* genes can be part of the molecular mechanism associated with this phenomenon, and in fact, DNA damage induces the expression of *FAS* in other cancer cells [50]. On the contrary, the treatment with rapamycin induces a clear increase in cell death, and an increase in the levels of cleaved caspase 3 and fragmented PARP, which suggests the initial steps of the apoptotic process.

Both drugs provoke a decrease in proliferative markers, such as *KI67 and PCNA*. It is well described that rapamycin can attenuate mammalian cell proliferation, including cancer and immune cells [51]. Inhibition of mTOR in B cells, with rapamycin, suppress cell proliferation by blocking cell cycle progression at G1 phase [52, 53]; indeed, mTOR is a crucial regulator of cell cycle progression from G1 to S phase [54].

Summarizing, etoposide and rapamycin have important effects on the metabolic gene expression pattern as well as apoptotic and proliferation markers in these cells. This work provides important evidence since the understanding of cancer metabolism is essential for clinical treatment using those drugs in future.

## Conflict of interest

The authors declare no conflict of interest.

## Author contributions

MC‐G and AZ conceived and designed the experiment; MC‐G, YA, CC, CM, PO, GV, and AZ performed experiments and acquired the data; MC‐G, CC, MS, FJM, and AZ analyzed and interpreted the data; MC‐G, MS, FJM, AR, and AZ drafted, edited, and revised manuscript.

## Data Availability

The data that support the findings of this study are available from the corresponding author (angara.zambrano@uach.cl) upon reasonable request.

## Appendix

**Table A1:** Oligonucleotide primers for real‐time RT‐qPCR.

Primers	Sequence 5ʹ–3ʹ
36B4 Forward	TGGCAGCATCTACAACCCTGAAGT
36B4 Reverse	TGGGTAGCCAATCTGAAGACAGACA
ACSL3 Forward	ACACAAGGGCGCATATCTTC
ACSL3 Reverse	GTGGTGTGACACAGGACCAG
BAX Forward	GGCAGCTGACATGTTTTCTGAC
BAX Reverse	CACCCAACCACCCTGGTCTT
BCL2 Forward	TTGAGTTCGGTGGGGTCAT
BCL2 Reverse	GACTTCACTTGTGGCCCAG
FAS Forward	AGCTTGGTCTAGAGTGAAAA
FAS Reverse	GAGGCAGAATCATGAGATAT
G6PD Forward	AAGAACGTGAAGCTCCCTGA
G6PD Reverse	AATATAGGGGATGGGCTTGGG
GLUT1 Forward	CTTCACTGTCGTGTCGCTGT
GLUT1 Reverse	CCAGGACCCACTTCAAAGAAA
GLUT3 Forward	GCTATGGCCGCTGCTACTGGG
GLUT3 Reverse	CCACAACCGCTGGAGGATCTGC
HIF1 Forward	CGTTCCTCCGATCAGTTGTC
HIF1 Reverse	TCAGTGGTGGCAGTGGTAGT
HK2 Forward	CAAAGTGACAGTGGGTGTGG
HK2 Reverse	GCCAGGTCCTTCACTGTCTC
KI67 Forward	CGTCCCAGTGGAAGAGTTGT
KI67 Reverse	CGACCCCGCTCCTTTTGATA
LDH Forward	ATCTTGACCTACGTGGCTTGGA
LDH Reverse	CCATACAGGCACACTGGAATCTC
MCM2 Forward	ATCTACGCCAAGGAGAGGGT
MCM2 Reverse	GTAATGGGGATGCTGCCTGT
mTAF Forward	AATGGATAGGCACAGGAAACC
mTAF Reverse	CAAGTATTATGCTGGCAGAAGTC
MKV Forward	GCTCAAGTTCCCAGAGATCG
MKV Reverse	ATGGTGCTGGTTCATGTCAA
NOXA Forward	GTGTTCCTGTTGGGCGTTAC
NOXA Reverse	GGATTTTCCGAACCTT
PCNA Forward	CCGGTTACTGAGGGCGAGAA
PCNA Reverse	GACGACCGGCTGAGACTTG
PDH Forward	GTGATAAATATGCGTACCATTAGACC
PDH Reverse	CAGCACCAGTGACACGAACAGC
PDK1 Forward	GAAGCAGTTCCTGGACTTCG
PDK1 Reverse	ACCAATTGAACGGATGGTGT
PFKP Forward	CTACAAGCGACTTGCCATCA
PFKP Reverse	ATCATAGATGGCGAGCATCCC
PGD Forward	GGTGCACAACGGGATAGAGT
PGD Reverse	CCATCGGTGTCTTGGAACTTT
PUMA Forward	GACCTCAACGCACAGTACGAG
PUMA Reverse	AGGAGTCCCATGATGAGATTGT
RPE Forward	TGGAAAGGATCTGGGAAGTG
RPE Reverse	CCTTGGGGTCAAGATCCATA
RPIA Forward	CTGGATCGACACCCAGAGAT
RPIA Reverse	CGATCACGATGAAGCGACTA
SC5D Forward	TATCTCTTCCGCCCATGTTC
SC5D Reverse	TGGCTCATTCACCATTTCAAA
SCD1 Forward	CCCAGCTGTCAAAGAGAAGGG
SCD1 Reverse	CAAGAAAGTGGCAACGAACA
TCL1 Forward	CGATACCGATCCTCAGACTCCAGTT
TCL1 Reverse	AAAGGAGACAGGTGCTGCCAAG
